# Mathematical Modeling for Removing Border Entry and Quarantine Requirements for COVID-19, Vanuatu

**DOI:** 10.3201/eid2805.211757

**Published:** 2022-05

**Authors:** Caroline van Gemert, Len Tarivonda, Posikai Samuel Tapo, Sereana Natuman, Geoff Clark, Joanne Mariasua, Nick Scott, Adam Craig, Myriam Abel, Matthew J. Cornish, Margaret Hellard, Rachel Sacks-Davis

**Affiliations:** The University of Melbourne, Carlton, Victoria, Australia (C. van Gemert);; Vanuatu Health Program, Port Vila, Vanuatu (C. van Gemert, G. Clark);; The Burnet Institute, Melbourne, Victoria, Australia (C. van Gemert, N. Scott, M. Hellard, R. Sacks-Davis);; Ministry of Health, Iatika Complex, Port Vila (L. Tarivonda, P.S. Tapo, S. Natuman, J. Mariasua);; Oxford University, Oxford, UK (N. Scott); University of New South Wales, Sydney, New South Wales, Australia (A. Craig);; World Health Organization, Port Vila (M. Abel); Dokta Blong Mi, Port Vila (M.J. Cornish)

**Keywords:** COVID-19, mathematical modeling, border entry, quarantine requirements, prevention, importation, coronavirus disease, respiratory infections, travel medicine, zoonoses, Vanuatu

## Abstract

The Pacific Island country of Vanuatu is considering strategies to remove border restrictions implemented during 2020 to prevent imported coronavirus disease. We performed mathematical modeling to estimate the number of infectious travelers who had different entry scenarios and testing strategies. Travel bubbles and testing on entry have the greatest importation risk reduction.

Many Pacific Island Countries and Territories (PICTs) implemented border entry restrictions and mandatory quarantine in 2020 to prevent imported coronavirus disease (COVID-19). Although some PICTs have experienced large-scale community transmission of COVID-19 (such as Fiji, Papua New Guinea, French Polynesia, and Guam), many PICTs have not (as of January 2022) experienced community transmission, including Vanuatu. Since March 2020, Vanuatu (population 301,695) has restricted entry to citizens and residents and required all incoming travelers to a complete 14-day quarantine period (*1*). As of January 10, 2022, a total of 7 border cases have been reported among travelers in quarantine in Vanuatu, and no community transmission (*2*).

The government of Vanuatu is considering various strategies to remove border restrictions and quarantine, including opening borders, creating travel bubbles with neighboring point-prevalence countries, and restricting entry to vaccinated travelers. We performed mathematical modeling to estimate the expected number of infected arrivals expected for each of these scenarios and through different testing strategies. This modeling complements other modeling that assessed importation risks of COVID-19 with higher point prevalence in the origin countries (*3*) and different outcomes, such as the expected time delay associated with different scenarios (*4*).

We developed an individual stochastic model to estimate the potential number of infectious travelers who would arrive in Vanuatu. We modeled 3 border scenarios and 4 testing strategies (Table). The probability of a traveler being infected on entry into Vanuatu was assumed to be a function of the point prevalence in the country of origin and the distributions of latent, presymptomatic and infectious, and symptomatic (or asymptomatic) infectious periods and test sensitivity. We used point prevalence estimates based on the epidemiologic situation on July 19, 2021, for neighboring countries, including New Caledonia (<0.001%) and New Zealand (0.001%) (*5*).

**Table:** Characteristics considered in the model for removing border entry and quarantine requirements for coronavirus disease, Vanuatu

Characteristic	Description
Border opening scenarios	
Scenario 1	Open border with no restrictions
Scenario 2	Travel bubble with low point-prevalence neighboring countries
Scenario 3	Travel bubble with low point-prevalence neighboring countries plus vaccination for all incoming travelers
Testing strategies	
Test strategy 1	No testing
Test strategy 2	Testing on arrival only
Test strategy 3	Predeparture plus on arrival
Test strategy 4	Predeparture plus on arrival plus day 5 after arrival

We assumed that passengers returning with a positive pretravel test result did not travel, those tested on arrival isolated until results were provided, and those tested on day 5 were in the community for 6 days (including time for testing and provision of results). We simulated 10,000 infected travelers stochastically and used 1,000 bootstrap samples to estimate uncertainty intervals. We applied the model to 40,000 passengers (15% of the number of arrivals in 2019) (*6*) (Appendix). We did not include additional variables, such as group size, masking, and hygiene measures.

The number of infectious persons in the community decreased by 98%–99% when travel was restricted entry to persons from low point-prevalence countries, compared with no restrictions on the country of departure for travelers (Figure). The number decreased further, by 61%–63% for each testing strategy, when travel was further restricted to vaccinated travelers only. For all scenarios, the number of infectious persons in the community was inversely proportional to the number of tests conducted. The greatest decrease was observed for testing on arrival (compared with no testing), for which the number of infectious cases in the community decreased by 42%–44%. The proportional decrease was 10%–14% when predeparture plus arrival testing was included. Although adding day 5 testing (in addition to predeparture and on arrival testing) did not result in further decrease in the number infectious persons in the community, it did identify 56%–67% of cases after entry, which would enable contact tracing to reduce risk for onward transmission.

**Figure:**
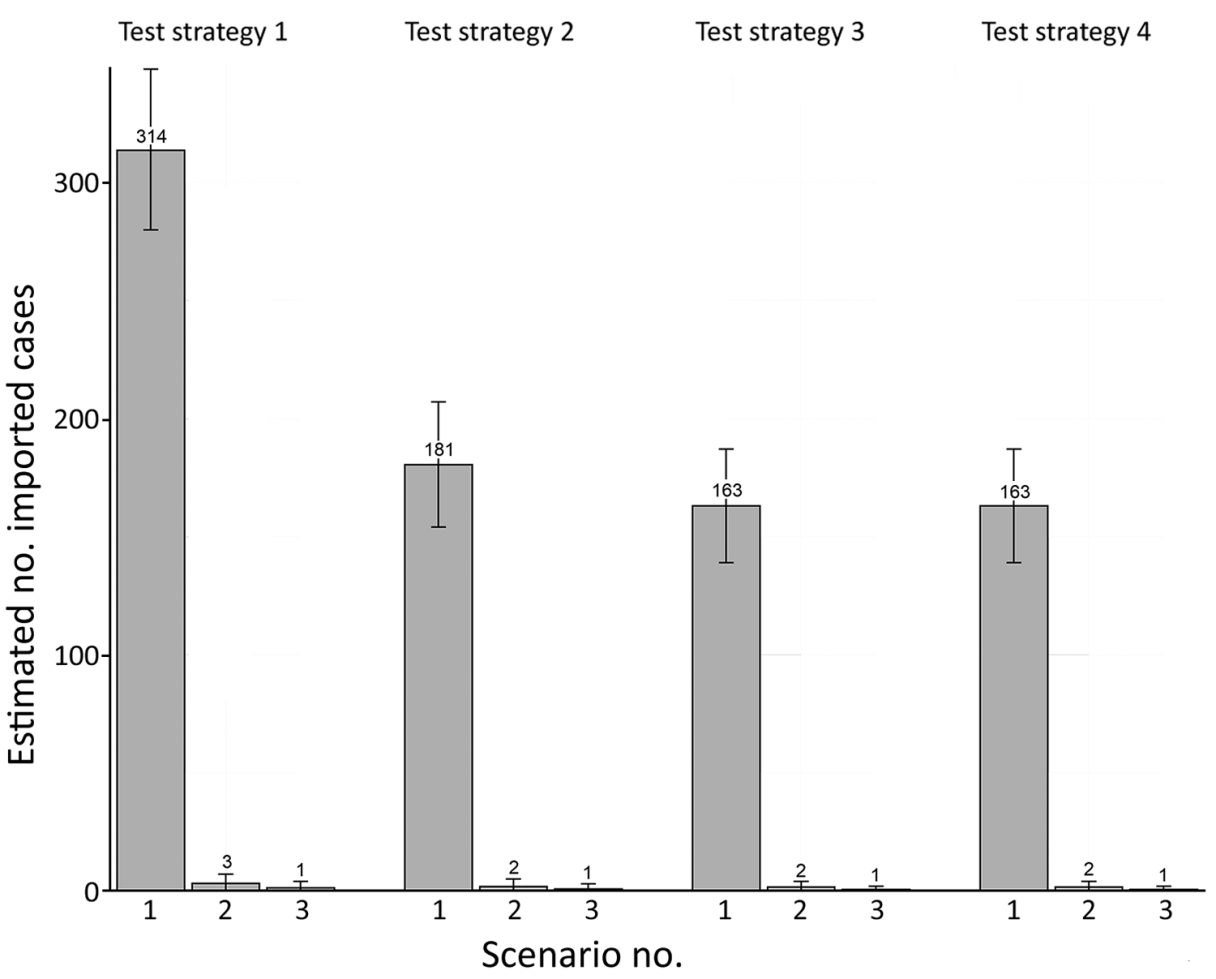
Number of imported cases of coronavirus disease in the community per 40,000 arrivals, by test strategy and epidemiologic scenario, Vanuatu. Error bars indicate 95% CIs.

Our analysis highlights that the scenario with the greatest importation risk reduction for Vanuatu is travel bubbles with low point-prevalence countries. The risk for case importation through quarantine-free travel with low COVID-19 incidence countries is <3.2 cases/40,000 travelers, an importation risk reduction of ≈100-fold compared with open borders. Several countries in the Pacific region have a low or zero COVID-19 point prevalence (*5*). Furthermore, country-level incidence might decrease as vaccination coverage increases because there is evidence that several COVID-19 vaccines might reduce transmission (*7*). On the basis of our results, many PICTs could be considered for quarantine-free travel with low risk for importation to Vanuatu.

Our results also demonstrate that COVID-19 testing on arrival is useful in all scenarios, but especially for open borders. Testing becomes increasingly useful as the point prevalence of COVID-19 increases in countries of travel origin. Testing 5 days after arrival enables detection of an additional 10%–14% of infections for all scenarios, and these cases can be contact traced and those infected quarantined for part of their infectious period. Since late 2020, Vanuatu has conducted arrival testing for all international arrivals (in addition to routine testing during quarantine). Our results confirm the usefulness of this strategy.

A limitation of our study is that the model does not estimate the number of secondary cases. Assumptions for parameters were based on published evidence for the original variant; these parameters might differ with new and emerging variants. In summary, as Vanuatu and other PICTs move toward removing border restrictions and importation prevention measures, on-arrival testing and restricting entry to travelers from low point-prevalence settings are essential strategies to limit COVID-19 cases.

Appendix Additional information on mathematical modeling for removing border entry and quarantine requirements for COVID-19, Vanuatu.
