# Supplementary material for: Mathematical Modeling for Removing Border Entry and Quarantine Requirements for COVID-19, Vanuatu
Source: Emerg Infect Dis. 2022 May;28(5):1053–5. doi: 10.3201/eid2805.211757 (PMC9045451; doi:10.3201/eid2805.211757)
Supplement: Appendix — Additional information on mathematical modeling for removing border entry and quarantine requirements for COVID-19, Vanuatu. [file 21-1757-Techapp-s1.pdf]

Article DOI: https://doi.org/10.3201/eid2805.211757

Mathematical Modeling for Removing Border Entry and Quarantine Requirements for COVID-19, Vanuatu

Appendix

Detailed Methods

An individual stochastic model of infection progression and recovery was used to estimate the risk for imported infection. A simulated sample of incoming travelers was simulated and analyzed in 3 steps:

Step 1: First, 10,000 persons infected with coronavirus disease (COVID-19) were simulated.

Step 2: These persons were sampled by using bootstrap methods to create 3 sets of 1,000 simulated populations of passenger arrivals to Vanuatu for analysis. Each of these populations included 40,000 persons; only a small minority of whom were infected with COVID-19 (0.004%–1% corresponding to ≈1.6–400 infected person/population, depending on the travel restriction scenario).

Step 3: Estimation of the COVID-19 importation risk based if the simulated persons were allowed to enter with no test, compared with scenarios including testing 72 hours before departure, on arrival, and 5 days after arrival.

Step 1: Simulated Population of 10,000 Infected Persons to Sample in Step 2

To simulate the sample of 10,000 infected persons (step 1), each person was assigned a time of infection, incubation period (time from infection to symptom onset), duration of infectiousness before symptom onset, and duration of infectiousness after symptom onset. Hypothetical severe acute respiratory syndrome coronavirus 2 nasopharyngeal swab specimen PCR results were simulated 72 hours before arrival, on arrival, and 5 days after arrival. Test sensitivity was assumed to vary by infectious stage (*1*) and symptom status (*2*): infectious stages

relevant to test sensitivity included the latent stage before infectiousness, presymptomatic, post-symptomatic and asymptomatic infectious stages. The test sensitivities within each stage were calibrated such that the distribution of test results in the infected population followed a similar distribution to the distribution described by Kucirka et al. for analysis of changes in PCR test sensitivity over time (*1*). Distributions for all parameters are summarized in Appendix Table 1.

Step 2: Creation of 3 Sets of 1,000 Simulated Populations of Passenger Arrivals to Vanuatu for Assessment of COVID-19 Importation Risk

Three sets of 1,000 simulated populations of passenger arrivals to Vanuatu were created. Each population of arrivals included 40,000 persons because this was the expected number of international arrivals to Vanuatu in the first year after borders reopened. The number of arrivals in the first year was estimated to be 15% of 2019 levels (n = 256,000) (*9*). Each set of 1,000 simulated populations was designed to capture a particular scenario regarding the probable point prevalence of 7 COVID-1 cases in the population of passenger arrivals (Appendix Table 2). The number of infected persons in each population of passenger arrivals within the set was assigned stochastically by using a Bernouli distribution, with the mean determined by the assumed point prevalence of COVID-19 among arrivals. For each population, infected persons were sampled from the 10,000 infected persons simulated in step 1.

Step 3: Estimation of COVID-19 Importation Risk by Travel Restriction and Testing Policy

The number of imported COVID-19 cases were calculated for each population simulated in Step 2 for each of the following 4 potential PCR testing policies:

1. No testing
2. Testing on arrival. Assume arrivals isolate until the negative result is received.
3. Preflight testing (72 hours before arrival) plus testing on arrival
4. Preflight testing, testing on arrival, and testing 5 days after arrival

Simulated persons were classified as having imported cases if they met the following criteria:

- They were infected in the 2 weeks before arrival in Vanuatu
- At least part of their infectious period was after arrival in Vanuatu

• They were not tested or had a negative test result during preflight and arrival testing

• For those who tested positive 5 days after arrival, part of their infectious period was before the time of the test.

References

1. Kucirka LM, Lauer SA, Laeyendecker O, Boon D, Lessler J. Variation in false-negative rate of reverse transcriptase polymerase chain reaction–based SARS-CoV-2 tests by time since exposure. Ann Intern Med. 2020;173:262–7. PubMed https://doi.org/10.7326/M20-1495

2. He D, Zhao S, Lin Q, Zhuang Z, Cao P, Wang MH, et al. The relative transmissibility of asymptomatic COVID-19 infections among close contacts. Int J Infect Dis. 2020;94:145–7. PubMed https://doi.org/10.1016/j.ijid.2020.04.034

3. Sah P, Fitzpatrick MC, Zimmer CF, Abdollahi E, Juden-Kelly L, Moghadas SM, et al. Asymptomatic SARS-CoV-2 infection: a systematic review and meta-analysis. Proc Natl Acad Sci U S A. 2021;118:e2109229118. PubMed https://doi.org/10.1073/pnas.2109229118

4. Lauer SA, Grantz KH, Bi Q, Jones FK, Zheng Q, Meredith HR, et al. The incubation period of coronavirus disease 2019 (COVID-19) from publicly reported confirmed cases: estimation and application. Ann Intern Med. 2020;172:577–82. PubMed https://doi.org/10.7326/M20-0504

5. He X, Lau EH, Wu P, Deng X, Wang J, Hao X, et al. Temporal dynamics in viral shedding and transmissibility of COVID-19. Nat Med. 2020;26:672–5. PubMed https://doi.org/10.1038/s41591-020-0869-5

6. Ferretti L, Wymant C, Kendall M, Zhao L, Nurtay A, Abeler-Dörner L, et al. Quantifying SARS-CoV-2 transmission suggests epidemic control with digital contact tracing. Science. 2020;368:eabb6936. PubMed https://doi.org/10.1126/science.abb6936

7. Wölfel R, Corman VM, Guggemos W, Seilmaier M, Zange S, Müller MA, et al. Virological assessment of hospitalized patients with COVID-2019. Nature. 2020;581:465–9. PubMed https://doi.org/10.1038/s41586-020-2196-x

8. Bullard J, Dust K, Funk D, Strong JE, Alexander D, Garnett L, et al. Predicting infectious SARS-CoV-2 from diagnostic samples. Clin Infect Dis. 2020;71:2663–6. PubMed https://doi.org/10.1093/cid/ciaa638

9. Vanuatu National Statistics Office. Statistics update: international visitor arrivals. December 2020 Provisional Highlights. February 10, 2021 [cited 2022 Feb 15]. https://www.stats.govt.nz/information-releases/international-travel-december-2021

10. World Health Organization. WHO coronavirus (COVID-19) dashboard 2021 [cited 2021 Jul 18]. https://covid19.who.int/

11. Pritchard E, Matthews PC, Stoesser N, Eyre DW, Gethings O, Vihta KD, et al. Impact of vaccination on new SARS-CoV-2 infections in the United Kingdom. Nat Med. 2021;27:1370–8. PubMed https://doi.org/10.1038/s41591-021-01410-w

12. Pouwels KB, Pritchard E, Matthews PC, Stoesser N, Eyre DW, Vihta K-D, et al. Effect of Delta variant on viral burden and vaccine effectiveness against new SARS-CoV-2 infections in the UK. Nat Med. 2021;27:2127–35. https://doi.org/10.1038/s41591-021-01548-7

Appendix Table 1. Distributions of key parameters

Variable	Distribution	Parameters	Reference
Asymptomatic infection	Bernouli	0.35	(*3*)
Timing of infection event before arrival	Uniform	0–14	Assumed
Time from infection event to symptoms	Lognormal	5.5, 2	(*4*)
Duration of presymptomatic infectious period	Lognormal	1,1	(*5*,*6*)
Duration of symptomatic infectious period	Lognormal	6,2	(*7*,*8*)
Test sensitivity before infectiousness	Bernouli	1 – test specificity	(*1*)*
Test sensitivity (late latent and early symptomatic period, symptomatic)	Bernouli	0.8	(*1*)*
Test sensitivity (late latent and early symptomatic period, asymptomatic)	Bernouli	0.6	Expert opinion and (*2*)
Test specificity	Bernouli	0.995	Expert opinion

*Infection period specific test sensitivities were calibrated such that the distribution of test results in the infected population followed a similar distribution to the distribution described by Kucirka et al. (*1*) for analysis of changes in PCR test sensitivity over time.

Appendix Table 2. Three scenarios modeled regarding travel restrictions and associated prevalence of coronavirus disease in the population of passenger arrivals

Travel restriction scenario	Assumed point prevalence, %	Source of estimate
No restrictions	1	Prevalence of coronavirus disease in arrivals to Australia, May–June 2020
Travel bubble	0.01	Prevalence of coronavirus disease in neighboring countries of New Caledonia (<0.001%) and New Zealand (0.001%) in July 2021 (*10*)
Travel bubble (vaccinated passengers only)	0.004	60% reduction in prevalence due to vaccination (*11,12*)
